# Pesticides Are an Occupational and Public Health Issue

**DOI:** 10.3390/ijerph15081650

**Published:** 2018-08-03

**Authors:** Hans-Peter Hutter, Hanns Moshammer

**Affiliations:** Department of Environmental Health, Medical University of Vienna, 1090 Vienna, Austria; hans-peter.hutter@meduniwien.ac.at

Concern regarding the health effects of pesticide residues in our food chain is rife. Working in environmental health, civil society representatives, policy makers, and concerned citizens often confront us requesting clear guidance on this topic. The safety of pesticide residues within the food chain has been studied (e.g., [1,2,3]), and evidence suggests that consumers’ diet leads to high levels of pesticides in breast milk [4]. If breast milk were to be sold in the supermarket, this would often go against current rules of food quality. This is an area of significant concern, particularly as contaminated breast milk poses a direct risk to the child [5].

Reports of contaminated breast milk as a result of pesticides may result in mothers seeking alternative methods for infant feeding. Similar concerns have been raised about potential pesticide contamination, e.g., of some types of fish [4]. Indeed, contamination of food, and especially of our most valuable types of food, is a public health concern and not only or not even predominantly because of direct risks of toxicity. There has been significantly less attention given to the other end of the food production line. Many foods products consumed in high-income countries are produced in low- and middle- income countries. Occupational conditions and pesticides exposure in these countries have received less attention and thus have rarely been studied [6,7]. Therefore, this special issue focusses on occupational pesticide exposures, particularly in the Global South.

We were successful in inviting submissions tackling our primary goal [8,9,10,11]. Pesticides per definition are toxic to “pests”, i.e., to plants or animals. Therefore, it is unsurprising that pesticides may also have toxic effects on “good guys”, including humans. The application and use of pesticides are therefore inherently prone to cause risk to human health. Legal frameworks exist to ensure that “proper use” of pesticides poses no grave risk to human health. However, experience from field studies indicates that “proper use” is a very fluid and vague term when implemented in real life contexts. Pesticides, through occupational exposure primarily in agricultural settings in conditions of poor socio-economic and educational status, have been linked to poor health outcomes. This includes cancer [10,12], respiratory disease [13], neurological diseases [14], developmental problems [15], and cardiovascular disorders [16].

The studied occupational settings are prone to exposures to multiple pesticides and a wide range of agro-chemicals [17,18]. Challenging, hard outdoor work within hot and semi-arid conditions may also contribute to adverse health outcomes [19,20]. Under such conditions, industry often successfully argues that there is no hard “proof” that any specific pesticide is responsible for observed increases in a multitude of diseases [21]. Therefore, mechanistic studies come in handy to document causal pathways leading to disease [22,23,24].

Both public and environmental health concerns, and occupational risks associated with pesticides, are areas of scientific and policy interest. There is, however, overly high concern and emotion associated with the consumption of potentially contaminated products, with relatively little attention paid to the exposure of producers. We hope that this special issue will encourage people to also focus on the health of producers. Pesticide exposure under conditions of insufficient workers’ protection is an important aspect that deserves increased (research) attention. 
